# 1,1-Bis(3'-indolyl)-1-(*p*-biphenyl)methane inhibits basal-like breast cancer growth in athymic nude mice

**DOI:** 10.1186/bcr1761

**Published:** 2007-08-31

**Authors:** Yunpeng Su, Kathryn Vanderlaag, Courtney Ireland, Janelle Ortiz, Henry Grage, Stephen Safe, Arthur E Frankel

**Affiliations:** 1 Scott & White Cancer Research Institute, South Airport Road, Temple, Texas 76502, USA; 2 Department of Veterinary Physiology & Pharmacology, Texas A&M University, 4466 TAMU, College Station, TX 77843-4466; 3 Plantacor, Inc., 526 University Dr. East Suite 101A, College Station, Texas 77840 USA

## Abstract

**Introduction:**

1,1-Bis (3'-indolyl)-1-(p-biphenyl) methane (CDIM9) has been identified as a new peroxisome proliferator-activated receptor (PPAR)-γ agonist that exhibits both receptor dependent and independent antitumor activities. CDIM9 has not previously been studied with respect to its effects against basal-like breast cancer. Our goal in the present study was to investigate the anti-basal-like breast tumor activity of CDIM9 *in vitro *and *in vivo*.

**Methods:**

The effects of CDIM9 on cell protein and DNA syntheses were determined in basal-like breast cancer MDA-MB231 and BT549 cells *in vitro*. Maximum tolerated dose and dose-limited toxicity were determined in BalB/c mice, and antitumor growth activities were assessed in MDA-MB231 basal-like breast tumor xenografts in athymic nude mice.

**Results:**

CDIM9 exhibited selective cell cytotoxicity and anti-proliferation effects on basal-like breast cancer lines. In MDA-MB231 cell, CDIM9 induced caveolin-1 and p27 expression, which was significantly downregulated by co-treatment with the PPAR-γ antagonist GW9662. Nonsteroidal anti-inflammatory drug-activated gene-1 and activating transcription factor-3 were upregulated by CDIM9 through a PPAR-γ independent pathway. CDIM9 (40 mg/kg daily, intraperitoneally, for 35 days) inhibited the growth of subcutaneous MDA-MB231 tumor xenografts by 87%, and produced a corresponding decrease in proliferation index. Nearly half of the treated mice (46%) had complete durable remissions, confirmed by histology. The growth of an established tumor was inhibited by CDIM9 treatment (64 mg/kg daily, intraperitoneally, for 10 days), with a mean tumor growth inhibition of 67% as compared with controls. CDIM9 induced increases in tumor caveolin-1 and p27 *in vivo*, which may contribute to its antitumor activity in basal-like breast cancer.

**Conclusion:**

CDIM9 showed potent antiproliferative effects on basal-like breast cancer cell in tissue culture and dramatic growth inhibition in animal models at safe doses. These findings justify further development of this drug for treatment of basal-like breast cancer.

## Introduction

Over 40,000 women each year in the USA are diagnosed with basal-like breast carcinoma [1]. This represents 20% of all breast cancers [2]. Basal-like breast cancers exhibit low expression of HER2 and estrogen receptor (ER), and high expression of epidermal growth factor receptor and cytokeratin-5/6 [3]. In addition, tumor cells often express mutant p53, or the exhibit *BRCA1 *mutations or gene silencing [4]. Patients with basal-like breast tumors are more likely to be African-American, to be premenopausal, and to have tumors with high nuclear grade, high histologic grade, high mitotic index, and unfavorable histology. Survival of these patients is poor, with twice the mortality of luminal breast cancer patients [1]. Unlike other breast cancers, there is no approved molecular targeted therapy, and therefore development of an effective agent remains an important goal in the treatment of basal-like breast carcinoma.

The gene expression profile of basal-like breast cancer is distinct from that of other subtypes of breast cancer. Many of the basal-like gene products have been implicated in cell proliferation, apoptosis regulation, and extracellular matrix remodeling [5]. Among the genes selectively altered in basal-like breast cancer are those encoding p27 and caveolin-1. The cell cycle inhibitor p27 inhibits cyclin-E/cyclin-dependent kinase-2, which prevents the activation of S-phase-specific transcription factors such as elongation factor-2. Cells become arrested in the G_1 _phase of the cell cycle [6]. In basal-like tumors, p27 expression is downregulated [7]. Caveolin-1, a 22 kDa protein, participates in caveolae formation and binds and inactivates cell surface protein kinases through its caveolar scaffolding domain (residues 82 to 101) [8]. Caveolin-1 expression is reduced in early mammary carcinogenesis [9], but increased levels have been found in many basal-like breast cancers [10]. Distinct domains of caveolin-1 (phosphorylated Tyr-14 and Ser-80 or mutated Pro-132) may override the growth inhibitory activity of the caveolin-1 and lead to tumor cell invasion and metastases [11].

We sought to define key regulatory genes that may modulate both p27 and caveolin-1 expression in basal-like tumor cells, and one such candidate is the peroxisome proliferator-activated receptor (PPAR)-γ. This critical transcription factor plays a role in a variety of biologic processes, including metabolism, inflammation, cell growth and differentiation, and there are reports that PPAR-γ is over-expressed in multiple tumor types and their derived cancer cells [12-14]. PPAR-γ is also expressed in the breast tumor derived cancer cell lines MDA-MB-231, MCF-7, SKBR-3, MDA-MB-435, and MDA-MB-453, irrespective of ER, HER2/neu, or p53 status [13,15,16]. Small molecule ligands bind PPAR-γ and form heterodimers with retinoid X receptors. The PPAR-γ/retinoid X receptor complex binds peroxisome-proliferation response element within promoters of target genes, recruits co-factor complexes (either co-activator or co-repressors), and then modulates their expression. PPAR-γ regulates expression of several genes in cancer cells lines, including p27 and caveolin-1 [17,18].

A number of PPAR-γ agonists have been tested preclinically and clinically, yielding evidence for tumor growth inhibition and differentiation in liposarcoma and prostate cancer [19,20]. The influences of other members of PPAR family on tumor growth are less investigated. PPAR-α agonists LY-171883 and WY-14,643 inhibit cyclo-oxygenase-2 and vascular endothelial growth factor transcriptional activation in human colorectal carcinoma cells via inhibition of activator protein-1 [21]. Fenofibrate decreases metastatic potential of melanoma cells *in vitro *via downregulation of Akt, and it inhibits melanoma tumor growth *in *vivo [22,23]. In breast cancer, however, one study suggests that PPAR-a activation increases proliferation of both MDA-MB-231 and MCF-7 cells [24]. The promotion of proliferation following PPAR-a activation is in stark contrast to the effects of PPAR-γ-activating ligands, which decrease proliferation in those cells [24].

The 1,1-bis(3'-indolyl)-1-(*p*-substituted phenyl)methanes containing *p*-trifluoromethyl, *p*-tbutyl, or *p*-phenyl (CDIM9) substituents were initially identified as a novel class of PPAR-γ agonists in breast cancer cells [25]. These compounds increase PPAR-γ activity in prostate, pancreatic, colon, and bladder cancer cells [26-33]. Similar to other PPAR-γ agonist, CDIM9 and related compounds exhibit a broad spectrum of anticancer activities by inducing cancer cell differentiation, growth inhibition, and apoptosis. The growth inhibition of cancer cells by CDIMs may be either PPAR-γ dependent or independent [26,27,33]. We chose to investigate anti-basal-like breast cancer activity of CDIM9 *in vitro *and *in vivo *and correlate the effects with modulation of PPAR-γ activity.

## Materials and methods

### Cells

The human basal-like breast cancer cell lines MDA-MB-231 and BT549 were purchased from the American Type Culture Collection (Manassa, VA, USA). MDA-MB231 cells were cultured in Leibovitz's L-15 medium with 2 mmol/l L-glutamine and 10% fetal bovine serum, at 37°C and 100% air. BT549 cells were cultured in RPMI 1640 medium with 2 mmol/l L-glutamine adjusted to contain 1.5 g/l sodium bicarbonate, 4.5 g/l glucose, 10 mmol/l HEPES, and 1.0 mmol/l sodium pyruvate supplemented with 0.023 IU/ml insulin and 10% fetal bovine serum. The primary human skeletal muscle cells (SKMCs) and human muscle myoblast (HSMM) were purchased from Lonza (Baltimore, MD, USA). SKMCs were cultured in SkGM SingleQuots medium (Lonza, Baltimore, MD, USA). with supplements and growth factors (human epidermal growth factor, insulin, bovine serum albumin, fetuin, dexamethasone, and gentamicin/amphotericin-B). HSMMs were cultured in SkGM-2 SingleQuots medium with supplements and growth factors (human epidermal growth factor, dexamethasone, L-glutamine, fetal bovine serum, and gentamicin/amphotericin-B) at 37°C and 5% carbon dioxide.

### Drug

CDIM9 used in this study was prepared using the same protocol as described previously [34], modified by Plantacor Inc. (Bryan-College Station, TX, USA). Briefly, indole was condensed with a *p*-phenyl substituted benzaldehyde at pH 2.5 in dilute aqueous acetic acid. The progress of the condensation reaction was monitored by thin-layer chromatography and, when 80% to 90% of the reaction was completed, the resulting solid was filtered and crystallized from 1-propanol. CDIM9 structure was confirmed by gas chromatography/mass spectrometry and/or nuclear magnetic resonance spectroscopy. The placebo used in this study contains 5% oleic acid, 0.2% vitamin E, 92.8% Cremophor ELP (^3^Plantacor College Station, TX, USA).

### Animals

Female Balb/c and athymic nude mice (nu^-/-^), aged 4 to 6 weeks, were purchased from Charles River Laboratories (Wilminton, MA, USA) and maintained in a ventilated rack system. Irradiated food and autoclaved water were provided *ad libitum*. These experiments were approved by the Institutional Animal Care and Use Committee at the Scott & White Memorial Hospital (Temple, TX, USA). The mice were allowed to adjust to their environment for 1 week before initiation of the experiments.

### Cytotoxicity assay and TUNEL staining

Cytotoxicity was determined using a [^3^H]leucine incorporation inhibition assay. Cells were plated in 96-well flat bottomed plates at 10^4 ^cells per well and cultured in the growth medium overnight. The next day, fresh medium was replaced containing serial diluted CDIM9 between 1 × 10^-3 ^mol/l and 1.9 × 10^-6 ^mol/l and cultured at 37°C for 48 hours. Then, 1 μCi (0.037 MBq) of [^3^H]leucine (NEN DuPont, Boston, MA, USA) in 100 μl RPMI-1640 leucine-free medium was added to each well to replace the old medium and incubation was continued for an additional 18 hours at 37°C/5% carbon dioxide. Cells were then harvested using a Skatron Cell Harvester (Skatron Instruments, Lier, Norway) onto glass fiber mats, and the counts/min of incorporated radiolabel was measured using an LKB-Wallac 1205 Betaplate liquid scintillation counter (Perkin-Elmer, Gaithersburg, MD, USA) gated for ^3^H. The percentage maximal [^3^H]leucine incorporation was then plotted versus the log of CDIM9 concentration, and nonlinear regression with a variable slope sigmoidal dose-response curve was generated along with EC_50 _(concentration producing 50% of the maximum possible response) using GraphPad Prism software (GraphPad Software, San Diego, CA, USA). All assays were done at least twice with an interassay range of 30% or less for EC_50_. Apoptosis was visualized with terminal deoxynucleotidyltransferase-mediated dUTP nick-end labeling (TUNEL) using the Apoptag kit (Serologicals, Norcross, GA, USA).

### Cell proliferation assay

MDA-MB231 and BT549 cells (2 × 10^4^) were plated in 12-well plates and allowed to attach for overnight. Fresh medium containing 1, 5, or 10 mmol/l of CDIM9 or solvent (Me_2_SO) was added every 48 hours and cells were then trypsinized and counted after 48, 96, 144 hours by trypan blue counting. Results for each treatment group were determined from triplicate studies.

### Maximum tolerated dose studies

To determine the maximum tolerated dose (MTS), to Balb/C mice per group were injected intraperitoneally with increasing doses of CDIM9 every day for a total 35 injections. Mice were monitored twice per day for sign of toxicity. Mice that exhibited dehydration, hypothermia, or dyspnea were considered moribund and were killed following institutional regulations. Samples from major organs were removed, fixed in 10% buffered formaldehyde, dehydrated, and embedding in paraffin. Sections were stained with hematoxylin and eosin and examined under a microscope. All surviving mice were killed at day 60 after injection.

### Antitumor efficacy studies

Athymic nude mice (Nu^-/-^) were injected intraperitoneally with 75 μg of a rat antimouse asialo GM1 antibody (Wako Chemical Company, Richmond, VA, USA) to reduce natural killer cells. Injections were carried out on days -4 and -2 before the injection of MDA-MB231 cells. At day 0, mice were injected subcutaneously in the left flank with 10^7 ^MDA-MB231 cells in 100 to 200 μl serum-free medium. Three groups of mice (12 to 13 mice/group) were then treated intraperitoneally with 40 mg/kg CDIM9 in 50 μl placebo, 50 μl placebo, or 50 μl phosphate-buffered saline (PBS) every day for 35 total injections starting at day 4 after tumor inoculations. Animals were observed twice daily and tumor sizes were measured twice per week using calipers, based on the formula L × W^2 ^(where L is the length and W is the width of the tumor). Moribund mice and mice whose tumor burdens exceeded 20% of their body weight were killed, as described above. All mice were killed at day 60 after tumor injection following institutional regulation. Tumor tissues were removed for immunohistochemistry staining and *in vitro *tumor cell cytotoxicity assay.

### Histological analysis

The organs (liver, spleen, heart, lung, kidney, small intestine, and brain) and tumors were fixed for 24 hours in 10% buffered formaldehyde, dehydrated, and embedded in paraffin. Sections were stained with hematoxylin and eosin, and subjected to microscopic analysis.

### Advanced tumor therapy with CDIM9

Thirty days after antitumor efficacy studies, four mice bearing tumors (average about 400 mm^3^) from the PBS-treated group were treated intraperitoneally with CDIM9 (64 mg/kg) or PBS every day for 10 total injections. On day 11, animals were killed following institutional regulations, and tumors were harvested and processed as described above. Proliferating cells were tested by staining of section with mouse anti-Ki-67 antibody (Neomarkers/Labvision) and angiogenesis was visualized by staining with mouse anti-CD34 antibodies (Neomarkers/Labvision, Fremont, CA, USA). Immunostaining was performed as described previously [35].

### Tumor tissue and cell lysate preparation and immunoblotting

The tumor tissue and cell lysates were prepared as described previously [36]. Briefly, tumor tissues were collected and kept frozen at -80°C until use. Ice cold lysis buffer (2% Triton X-100, 10 mmol/l Tris-HCl [pH 8], 150 mmol/l NaCl, 10 mmol/l NaN3, and 10 mmol/l EDTA) containing protease inhibitors (Sigma, St. Louis, MO, USA) was added to tissues (20 mg tissue/ml lysis buffer). Tissues were homogenized using a polytron and maintained on ice for 60 min. Soluble material was selected by centrifugation at 39,800 g for 30 min at 4°C using a J-17A rotor. Whole cell lysates were obtained using Ack lysing buffer with protease inhibitor cocktail. The tissue lysates were tested by Western blot with caveolin-1 (1:1,000 dilution), p21 (1:1,000), and p27 (1:200) antibodies (Santa Cruz Biotechnology Inc., Santa Cruz, CA, USA). Anti-β-actin was used at 1:1,000 dilution (Sigma). The peroxidase-conjugated antimouse or antirabbit antibodies were used at 1:1,000 dilution (Jackson ImmunoResearch, West Grove, PA, USA).

### Statistical analysis

Survival was analyzed using the Kaplan-Meier method. For comparison of tumor volumes and EC_50_s, a Mann-Whitney or Kurskal-Wallis test was used. *P *values below 0.05 were considered to represent statistical significance. Statistical analyses were conducted using GraphPad Prism software (GraphPad Software, San Diego, CA, USA). Statistical comparisons were conducted using a 5% level of significance.

## Results

### Cytotoxicity of CDIM9

**Figure 1:**
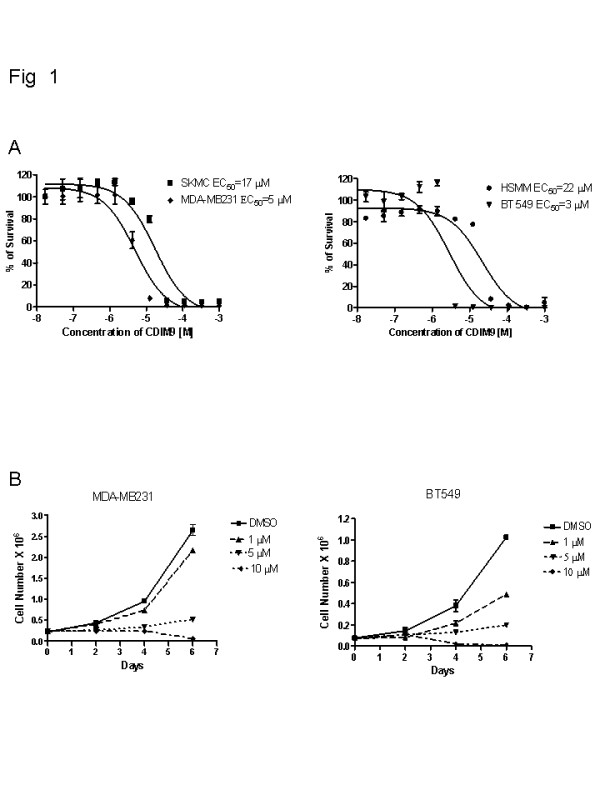
The cytotoxic and growth inhibitory effects of CDIM9 in MD-MB231 and BT549 cells. **(a) **MDA-MB231 cells, SKMCs, BT549 cells, and HSMMs were treated with serial diluted CDIM9 at between 1 × 10^-3 ^and 1.9 × 10^-6 ^mol/l for 48 hours. The EC_50 _values were determined using GraphPad Prism software. **(b) **Cell growth inhibition assay. MD-MB231 and BT549 cells were treated with 1 to 10 μmol/l CDIM9 for 6 days, and cell numbers were determined as described in Materials and methods. Results are expressed as means ± standard error for three separate determinations at each time point. CDIM9, 1,1-bis (3'-indolyl)-1-(p-biphenyl) methane; EC_50_, concentration producing 50% of the maximum possible response; HSMM, human muscle myoblast; SKMC, human skeletal muscle cell.

Two different cell cytotoxicity assays were performed. Protein synthesis inhibition assay revealed that that two different human basal-like breast cancer cells, namely MD-MB231 and BT549, exhibited comparable sensitivities to CDIM9, with EC_50 _values of 5 and 3 μmol/l, respectively, after treatment for 48 hours (Figure 1a). In comparison, SKMCs and HSMMs were less sensitive to CDIM9 than were tumor cells, with EC_50 _values of 17 and 22 μmol/l, respectively. A cell proliferation inhibition assay based on viable cell counts also showed that CDIM9 inhibited proliferation of the basal-like MDA-MB231 and BT549 cells, with 50% inhibitory concentrations values of 1 to 5 μmol/l (Figure 1b).

### Effects of CDIM9 on cell cycle related proteins

**Figure 2:**
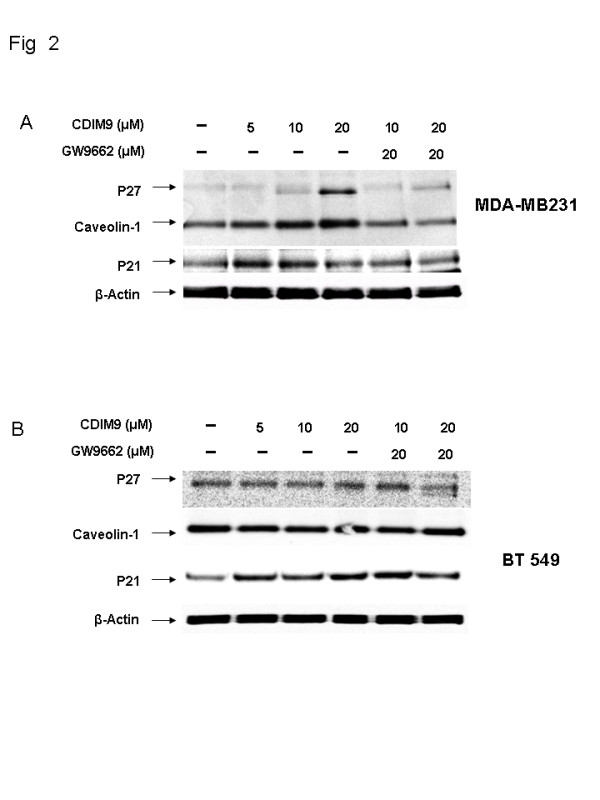
Induction of cell cycle proteins by CDIM9. **(a) **MDA-MB231 and **(b) **BT549 cells were treated with Me_2_SO or 5, 10, and 20 μmol/l CDIM9 for 24 hours. Whole cell lysates were analyzed for caveolin-1, p27, and p21 by Western blot assays, as described in Materials and methods. β-Actin served as loading control. CDIM9, 1,1-bis (3'-indolyl)-1-(p-biphenyl) methane.

The effects of CDIM9 on expression of various cell cycle proteins were investigated in MDA-MB231 and BT549 basal-like breast cancer cells treated with 5 to 20 μmol/l CDIM9 for 24 hours (Figure 2a). CDIM9 increased p27 expression level in MDA-MB231 cells in a concentration-dependent manner. Concentrations of 10 and 20 μmol/l of CDIM9 increased p27 expression 2.1-fold and 8-fold in MDA-MB231 cells, respectively (Figure 2a). The expression of caveolin-1 was also affected by CDIM9 treatment; in MDA-MB231 cells caveolin-1 expression was upregulated 1.7-fold, 2.4-fold, and 3.4-fold after treatment with 5, 10, and 20 μmol/l CDIM9, respectively (Figure 2a). Levels of p21 were not affected in MDA-MB231 cells but were slightly increased (1.5-fold) in BT549 cells treated with 5 to 20 μmol/l CDIM9. Neither p27 nor caveolin-1 was induced in BT549 cells after treatment with CDIM9 (Figure 2b).

To further investigate the role played by PPAR-γ in mediating CDIM9 induction of caveolin-1 and p27, MDA-MB231 cells were treated with 10 or 20 μmol/l CDIM9 in combination with 20 μmol/l of the PPAR-γ antagonist GW9662. Co-treatment of GW9662 inhibited the induction of caveolin-1 and p27 by CDIM9 in MDA-MB231 cells (Figure 2a), suggesting that these responses were PPAR-γ dependent, as previously described in colon and pancreatic cancer cells [26,28-30].

**Figure 3:**
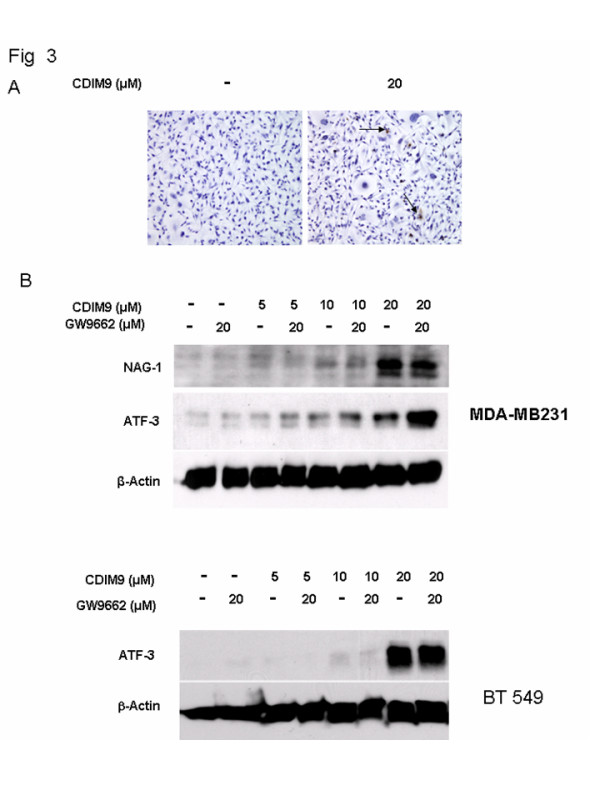
Effect of CDIM9 on apoptosis and induction of NAG-1 and ATF3. **(a) **MDA-MB231 cells were treated with Me_2_SO and 20 μmol/l CDIM9 for 24 hours and apoptotic cells were detected using TUNEL staining. The arrows indicated infrequent apoptotic cells. **(b) **MDA-MB231 and BT549 cells were treated with Me_2_SO or 5, 10, and 20 μmol/l CDIM9 for 24 hours. Whole cell lysates were analyzed for NAG-1 and ATF3 by Western blot assays. β-Actin served as loading control. ATF, activating transcription factor; CDIM9, 1,1-bis (3'-indolyl)-1-(p-biphenyl) methane; NAG, nonsteroidal anti-inflammatory drug-activated gene; TUNEL, terminal deoxynucleotidyltransferase-mediated dUTP nick-end labeling.

No significant induction of apoptosis was observed in MDA-MB231 cells treated with up to 20 μmol/l CDIM9 using TUNEL staining (Figure 3a). Furthermore, the pan-caspase inhibitor Z-VAD-FMK had no inhibitory effects on the cytotoxicity of CDIM9 in MDA-MB231 cells (data not shown). Based on observation of CDIM9 with other tumor cells [23,24], we analyzed the levels of the transcription factors nonsteroidal anti-inflammatory drug-activated gene (NAG)-1 and activating transcription factor (ATF)3 in the basal-like breast cancer cells treated and untreated with CDIM9. NAG-1 was upregulated in MDA-MB231 cells after treatment with 10 and 20 μmol/l CDIM9. ATF3 was also elevated in both MDA-MB231 and BT549 cells treated with CDIM9 (Figure 3b). The induction of NAG-1 and ATF3 were not inhibited by the PPAR-γ antagonist GW9662.

### Maximum tolerated dose and dose-limiting toxicity of CDIM9

**Figure 4:**
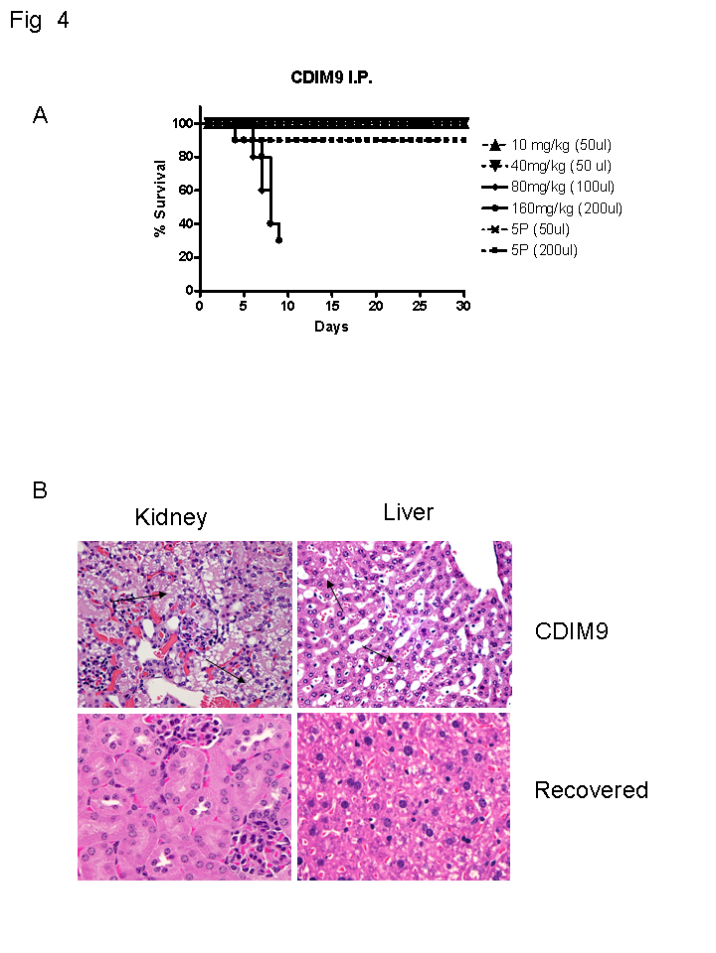
Maximum tolerated dose and dose limiting toxicity of CDIM9. **(a) **Kaplan-Meier curves for 10 BalB/c mice (age 4 to 6 weeks) injected intraperitoneally (i.p.) with CDIM9 daily for a total of 35 doses. **(b) **Hematoxylin and eosin staining of kidney and liver harvested from animals treated with 160 mg/kg per day CDIM9 for 12 days and after animals had recovered from treatment. Arrows indicated the tubular vacuolization (kidney) and steatosis (liver). CDIM9, 1,1-bis (3'-indolyl)-1-(p-biphenyl) methane.

As shown in Figure 4a, the MTD of CDIM9 given by intraperitoneal injection every day was 40 mg/kg. No deaths were observed at or below the MTD of all animals (two groups of 10). In contrast, animals that received 80 mg/kg and 160 mg/kg of drug for 10 continuous days exhibited mortality rates of 60% and 70%, respectively.

The dose-limiting toxicities of CDIM9 were kidney and liver damage. As shown in Figure 4b, intraperiteonal injections of 160 mg/kg CDIM9 for 10 continuous days caused 70% of animals to die. The remaining animals at this dose became moribund, with fur ruffling and loss of physical activity by day 10. Necropsies of dead or terminally ill animals showed profound histologic damage to kidney (tubular vacuolization) and liver (steatosis; Figure 4b). The infrequent apoptotic cells in the small intestine might be associated with the location for the injection of CDIM9 (data not shown). The heart, spleen, lung, and brain were not affected by CDIM9 treatment and showed no signs of histologic toxicity. Recovered mice from CDIM9 treatment after 3 weeks showed complete resolution of tubular vacuolization and hepatic steatosis (Figure 4b).

#### Human basal-like breast tumor growth in athymic nude mice.

Athymic nude mice treated with anti-asialo GM1 antibody were inoculated with 10^7 ^MDA-MB231 cells. After a long lag-phase, subcutaneous tumors exhibited rapid tumor growth. Mean ± standard error tumor volume was 336 ± 56 mm^3 ^after 29 days, which then doubled (672 ± 180 mm^3^) by day 35 and then again (1,129 ± 372 mm^3^) by day 39. The tumors continued to grow until animals were killed on day 43 (1,430 ± 462 mm^3^). Based on these results, we could detect a 50% tumor growth inhibition with 12 to 13 animals per group, respectively, with a two-sided type I error of 5% and a power 0.9. Pathology of the tumors confirmed the malignant histology.

### Inhibition of basal-like breast tumor growth by CDIM9

**Figure 5:**
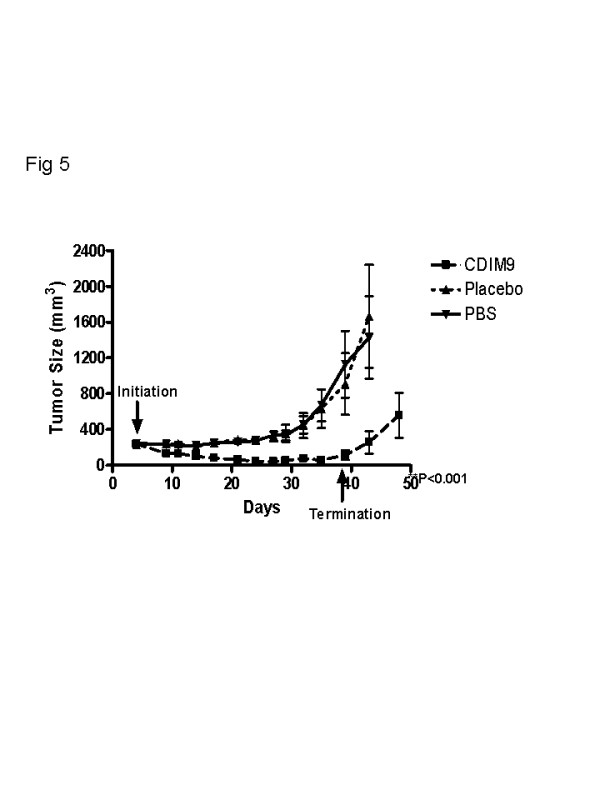
Inhibition of basal-like breast tumor growth by CDIM9. Shown are tumor sizes (following inoculation of MDA-MB231 basal-like breast cancer cells subcutaneously) tumor size in athymic nude mice treated daily with CDIM9 (40 mg/kg in 50 μl placebo, intraperitoneally), placebo, or PBS starting on day 4 after tumor inoculation. Values are expressed as mean ± standard error. CDIM9, 1,1-bis (3'-indolyl)-1-(p-biphenyl) methane; PBS, phosphate-buffered saline.

Beginning on day 4 after tumor cell inoculation, a cohort of 12 to 13 animals received treatment systemically by intraperitoneal injection with 40 mg/kg CDIM9, 50 μl placebo, or saline every day for a total of 35 doses (Figure 5). On day 29 the saline and placebo control tumor volumes were 336 ± 56 mm^3 ^and 359 ± 95 mm^3^, respectively. The volumes of CDIM9 treated tumor volumes were only 53 ± 148 mm^3 ^(*P *= 0.009 versus placebo control and *P *= 0.006 versus saline control). By day 39 when treatment was terminated, the saline treated tumor volumes were 1,129 ± 372 mm^3 ^and the placebo treated tumors were 910 ± 343 mm^3^. The CDIM9-treated tumor volumes were 115 ± 54 mm^3 ^(*P *= 0.009 versus placebo control and *P *= 0.006 versus saline control). By day 48, six out of 13 (46%) originally CDIM9-treated mice remained in complete remission both grossly and by histology. However, tumors in three mice re-grew to between 600 and 1,200 mm^3^.

### Growth inhibition of established tumor by CDIM9

**Figure 6:**
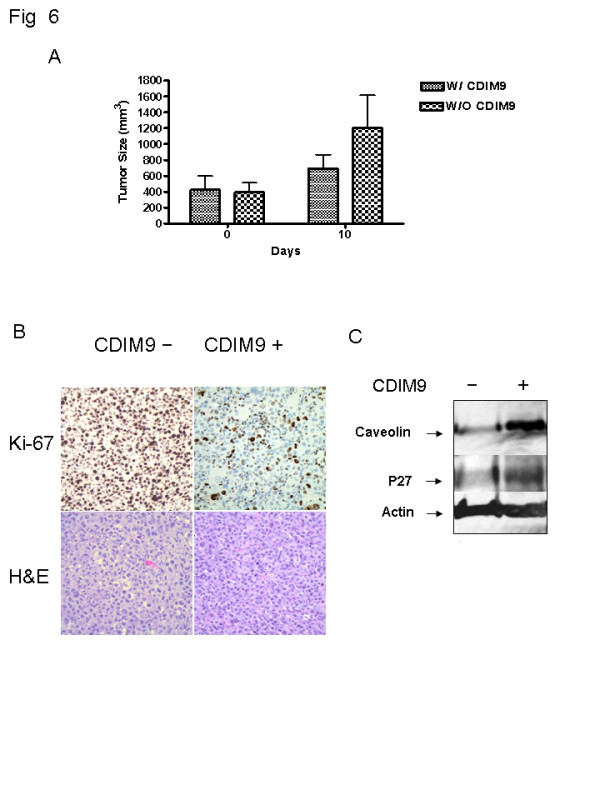
The growth inhibitory effects of CDIM9 on established MDA-MB231 tumor. **(a) **Athymic nude mice bearing established MDA-MB231 tumors were injected intraperitoneally with 64 mg/kg CDIM9 in 100 μl placebo or 100 μl saline for 10 total doses. The tumor size was measured on days 0 and 10. **(b) **Tumors collected from CDIM9 (64 mg/kg per day, intraperitoneally) or PBS treated mice were tested by Ki-67 immunohistochemistry staining and H&E staining. **(c) **The *in vivo *gene modulation activity of CDIM9 was investigated by immunoblotting analyses of MDA-MB231 tumor lysates after treatment with 64 mg/kg CDIM9 by intraperitoneal injection every day for seven total doses. CDIM9, 1,1-bis (3'-indolyl)-1-(p-biphenyl) methane; H&E, hematoxylin and eosin; PBS, phosphate-buffered saline.

We then evaluated the therapeutic efficacy of CDIM9 in established MDA-MB231 basal-like breast tumors. Nude mice bearing solid subcutaneous tumor nodules constituting 1.5% to 3% of the total body mass were treated with intraperitoneal injections of 64 mg/kg CDIM9 for 10 days (Figure 6a). The tumor growth was inhibited by CDIM9 treatment. By day 11 the treatment caused 67% tumor growth inhibition as compared with the size of tumor treated with saline (Figure 6a). Immunohistologic analysis of tumor tissues revealed inhibition of tumor cell proliferation with dramatic cession of Ki-67 staining (Figure 6b). Moreover, CDIM9 did not produce tumor endothelial cell damage based on equal CD34 vessel staining of CDIM9-treated tumor samples compared with PBS-treated tumor samples (data not shown).

### *In vivo *effects of CDIM9 on cell cycle-related proteins

The *in vivo *gene modulation activity of CDIM9 was further investigated by immunoblotting analyses of MDA-MB231 xenograft tumor lysate after treatment with 64 mg/kg CDIM9 by intraperitoneal injection every day for seven total doses. As shown in Figure 6c, a dramatic increase in caveolin-1 expression (7.2-fold) was detected in tumors from CDIM9 treated mice. Expression of p27 was moderately induced (2.9-fold) after CDIM9 treatment.

## Discussion

CDIM9 exhibited remarkable growth inhibitory effects on basal-like breast cancer in our animal model, with 87% tumor growth inhibition after daily treatment for 35 days. The dose used was twofold below the toxic dose of CDIM9; we therefore estimate a therapeutic index of at least 2, and this may be significantly higher because we did not investigate lower doses of CDIM9. In addition, six out of 13 (46%) of the tumor-bearing animals had complete tumor regression with no re-growth of tumors by day 50 and absence of basal-like breast tumor cells by histologic examination of necropsy specimens. This anticancer efficacy compares favorably with results obtained in similar subcutaneous MDA-MB231 xenografts in athymic nude mice treated with gamma radiation (94% tumor growth inhibition), doxorubicin (63% tumor growth inhibition), and paclitaxel (50% growth inhibition) [37-39]. Because each of the listed treatments are currently used for treatment of basal-like breast cancer and have demonstrated clinical benefit, we expect CDIM9 will also be beneficial in these patients.

Other PPAR-γ agonists with chemical structures different from that of CDIM9 have been tested *in vitro *and *in vivo *in basal-like and ER-positive breast tumors [16,40,41]. 2-Cyano-3, 12-dioxooleana-1, 9-dien-28-oic acid (CDDO) produced 60% tumor growth inhibition of MDA-MB435 cells in nude mice [16]. Troglitazone significantly inhibits MCF7 tumor growth (>85%) in triple-immunodeficient BNX nude mice [41]. A pilot study of short-term rosiglitazone therapy in early-stage breast cancer patients led to local and systemic effects on PPAR-γ signaling, but it did not show significant effects on breast tumor cell proliferation using Ki67 expression [40]. 15-Deoxy-delta12, 14-prostaglandin J_2 _(15dPGJ_2_) or troglitazone attenuated cellular proliferation of the ER-negative MDA-MB-231 cells, as well as the ER-positive line MCF-7. This was marked by a decrease in total cell number and by an inhibition of cell cycle progression [42]. Brief exposure of MDA-MB-231 cells to 15dPGJ_2 _inhibited tumorigenesis of MDA-MB231 cells in a nude mouse model [42]. 15dPGJ_2 _also induces cytotoxic effects in basal-like breast cancer cells, including MDA-MB231, BT549, and HS578T cells, through PPAR-γ independent mechanisms [43]. In MDA-MB231 cells, 15dPGJ_2 _increased expression of p21^Waf1/Cip1^, p27^Kip1^, and other *de novo *gene expressions [44]. CDDO transactivated PPAR-γ and induced dose-dependent and time-dependent cell growth inhibition, cell cycle arrest in G_1_-S and G_2_-M, and apoptosis in MDA-MB231 and MDA-MB435 cells [16]. We expect CDIM9 to exhibit improved anti-breast-cancer properties. Its PPAR-γ activities are stronger, and it has PPAR-γ independent tumor inhibitory properties. Furthermore, we intend to use this drug to treat patients with metastatic basal-like breast cancer.

The mechanism underlying tumor growth inhibition by CDIM9 probably involves PPAR-γ activation and upregulation of the cell cycle regulating genes p27 and caveolin-1. Tumor growth inhibition with CDIM9 *in vitro *and *in vivo *correlated with increased p27 and caveolin-1 protein expression. The induction of p27, but not that of caveolin-1, was dependent on the concentration of CDIM9 *in vitro*. The greater induction of caveolin-1 compared with p27 *in vivo *may reflect pharmacologic barriers preventing the drug from reaching all of the tumor cells *in vivo*. A member of KIP/CIP family of cyclin-dependent kinase inhibitors, p27 blocks G_1_-S cell cycle progression by binding and inhibiting cyclin-E/cyclin-dependent kinase-2 [45]. Levels of p27 are low in many breast cancers, in particular basal-like breast cancers [46]. The expression of p27 is upregulated by PPAR-γ agonists in some cancer cell lines [17], and the p27 promoter contains PPAR-γ response elements. Immunohistochemistry of breast tumors demonstrated linked expression of PPAR-γ and p27 [17,47]. Shortened survival of basal-like breast cancer patients was closely associated with decreases in nuclear p27 [7,47]. In our study, induction of p27 by CDIM9 was PPAR-γ dependent (based on PPAR-γ antagonist modulation). Furthermore, we and other groups have found that CDIM9 produces cell cycle arrest both in tissue culture and in animal tumors [25,48]. Caveolin-1 contains a caveolin scaffolding domain that inhibits activation extracellular signal-regulated kinase-1/2, represses cyclin D_1 _transcription, and induces CIP-dependent G_0_/G_1 _arrest [11]. There is preclinical and clinical evidence that caveolin-1 is a breast tumor suppressor gene [8,9,49]. PPAR-γ binds to response elements in the caveolin-1 promoter and triggers caveolin-1 transcription [18,50]. Although basal/mesenchymal MDA-MB231 cells contain measurable caveolin-1, PPAR-γ activation led to further increases in caveolin-1 expression and cell growth arrest. Our findings suggest that caveolin-1 may still possess tumor suppressor activities in basal-like breast cancers. Our combined results with p27 and caveolin-1 demonstrate a potential PPAR dependent molecular mechanism for the inhibition of tumor growth by CDIM9.

Because CDIM9 exhibits PPAR-γ dependent and independent effects on tumor cells [26,27,33], there are probably other factors that contribute to the observed inhibition of basal-like breast cancer growth. Our observation of ATF-3 and NAG-1 induction by CDIM9 may also contribute to tumor growth inhibition. Both of these transcription factors are negative regulators of tumor cell growth. ATF-3 inhibits cancer cell proliferation and invasion, and its induction correlates with cellular damage [51,52]. Interleukin-10 induced ATF-3 transcriptional suppression of matrix metalloproteinase-2 gene expression in human prostate CPTX-1532 cells [53]. Indole-3-carbinol and CDIM compounds induce proapoptotic gene NAG-1 expression mediated by ATF3 in human colorectal cancer cells [26-28,54]. Furthermore, ATF-3 is upregulated in HCT-116 human colorectal cancer cells following treatment with PPAR-γ agonist troglitazone, nonsteroidal anti-inflammatory drugs, diallyl disulfide, and resveratrol [55]. NAG-1, a member of the transforming growth factor (TGF)-β superfamily, inhibits proliferation of breast carcinoma cells [56], mink lung epithelial cells, and prostate carcinoma cells [57]. NAG-1 is induced by multiple agents including CDIM compounds [26-28]. Its induction can be either mitogen-activated protein kinase dependent (in LNCaP prostate cancer cells) or phosphoinositide-3 kinase dependent (in MDA-MB231 basal-like breast cancer cells and HCT-116 colon cancer cells). In MDA-MB231 cells, ATF-3 and NAG-1 are upregulated through a PPAR-γ independent pathway. In BT549 cells, the increase in ATF3 after CDIM9 treatment leads to the observed growth inhibition *in vitro*. We did not observe caspase dependent cytotoxicity or positive TUNEL staining in MDA-MB231 and BT549 cells after CDIM9 treatment. Other PPAR-γ agonists, including CDDO and troglitazone, yielded low percent apoptosis of MDA-MB231 and MDA-MB468 basal-like breast tumor cells [25,48].

ER-negative basal-like breast cancer cells such as MDA-MB231 are highly invasive and metastatic in rodent models. They are generally independent of exogenous hormone. Autocrine growth factors such as TGF-β are necessary for growth and survival of MDA-MB231 cells [58]. It has been reported that exogenous growth factors could influence the angiogenesis, metastasis, and tumorigensis of basal-like breast cancer cells. TGF-β enhances bone metastases in MDA-MB231 cells through stimulation of cyclo-oxgenase-2 expression [59]. Hepatocyte growth factor/scatter factor increases the invasiveness and migration of MDA-MB231 cells *in vitro *and induces angiogenesis [60]. Interleukin-1α also contributes to the local invasiveness and malignant behavior in less differentiated and ER-α negative tumors [61]. Tissue factor and factor VIIa promote MDA-MB231 tumor cells migration and invasion [62]. CDIM9 and its analogs were reported to inhibit tumor necrosis factor-α induced endothelial cell activation by inhibiting the expression of intercellular adhesion molecule-1, interleukin-6 and monocyte chemoattractant protein-1 [63]. The involvement of growth factors in basal-like breast cancer cell growth inhibition by CDIM9 will be explored in future.

Established tumors exhibited tumor growth inhibition, but there were no observed regressions. The lack of regressions in these tumors may relate to poor drug penetration or limited cytolytic toxicity. Our observations that relapsed tumor cells remained sensitive to CDIM9 are consistent with both pharmacologic explanations. Combinations of CDIM9 with other agents such as retinoic acid receptor ligands may produce greater anti-breast-cancer efficacy, as previously shown in preneoplastic rodent mammary treated with thiazolidinedione PPAR-γ agonists and all-trans-retinoic acid [64]. Alternatively, cytoreduction with chemotherapy may reduce tumor burden, facilitating control with CDIM9 monotherapy.

The dose limited toxicities of CDIM9 appeared to be hepatic steatosis and renal tubular vacuolization. The spleen, heart, lung, brain, and bone marrow were not affected by CDIM9 treatment based on histopathology. CDIM9 is a potent PPAR-γ agonist [25], and this receptor is a master regulator of adipogenesis and lipogenesis. The ligand-receptor complex coordinates transcription of multiple adipogenic and lipogenic genes, leading to lipid accumulation [65]. In the liver, lipid mediated stimulation of PPAR-γ leads to steatosis and liver injury [66]. PPAR-γ is also expressed in the kidney mesangial and tubular cells, and its activation can produce lipotoxicity, inhibition of cell proliferation, and cell death [67]. We only observed hepatic steatosis and renal tubular vacuolization in mice treated with CDIM9 at 80 or 160 mg/kg per day in the 12-day study. Furthermore, these toxicities were reversible after 3 weeks of recovery.

## Conclusion

In summary, CDIM9 exhibited potent antiproliferative effects on basal-like breast cancer cell in tissue culture and dramatic growth inhibition in animal models at safe doses. These results justify further development of this drug for therapy of basal-like breast cancer patients. We have prepared a clinical batch of CDIM9 for clinical studies, and the trials on metastatic basal-like breast cancer patients should be initiated by 2008.

## Abbreviations

ATF = activating transcription factor; CDDO = 2-cyano-3, 12-dioxooleana-1, 9-dien-28-oic acid; CDIM9 = 1,1-bis (3'-indolyl)-1-(p-biphenyl) methane; 15dPGJ_2 _= 15-deoxy-delta12, 14-prostaglandin J_2_; EC_50 _= concentration producing 50% of the maximum possible response; ER = estrogen receptor; HER = human epidermal growth factor receptor; HSMM = primary human muscle myoblast; NAG = nonsteroidal anti-inflammatory drug-activated gene; PBS = phosphate-buffered saline; PPAR = peroxisome proliferator-activated receptor; SMKC = human skeletal muscle cell; TGF = transforming growth factor; TUNEL = terminal deoxynucleotidyltransferase-mediated dUTP nick-end labeling.

## Competing interests

The authors declare that they have no competing interests.

## Authors' contributions

YS designed the experiments, performed the analysis and interpreted data, and drafted the manuscript. AEF critically reviewed all of the assays and revised the manuscript. KV and SS carried out NAG-1 and ATF-3 tests on cells treated with CDIM9 and critically revised the manuscript. CI carried out cytotoxicity determination and animal treatment. JO performed the Western blot and immunohistochemistry staining experiments. HG provided the CDIM9 drug. All authors read and approved the manuscript.
